# The characteristics of an effective clinical instructor from the perspective of nursing students: a qualitative descriptive study in Iran

**DOI:** 10.1186/s12912-021-00556-9

**Published:** 2021-03-04

**Authors:** Ali Soroush, Bahare Andaieshgar, Afsoon Vahdat, Alireza Khatony

**Affiliations:** Social Development and Health Promotion Research Center, Health Institute, Kermanshah University of Medical Sciences, Kermanshah, Iran

**Keywords:** Students, Perspective, Qualitative research, Nursing education research, Faculty

## Abstract

**Background:**

Clinical instructors have an important role in advancing nursing students to achieve the program objectives. Nursing student perceptions about the characteristics of an effective clinical instructors may help programs improve clinical instruction. As such, the purpose of this study was to understand the characteristics perceived by students to define an effective clinical instructor.

**Methods:**

In this qualitative descriptive study, semi-structured interviews were conducted with undergraduate and postgraduate nursing students. The sample was purposefully selected. MAXQDA software was used for the content analysis. The codes were organized into subcategories and consolidated into categories.

**Results:**

Data saturation was reached with twelve participants, including seven women and five men, between 21 and 36 years of age. General and special characteristics were the two main categories that emerged from the data. These categories were defined by nine subcategories including internal motivation, professional acceptability, clinical competency, teaching skill, clinical experience, values, being a faculty member, appropriate appearance, and communication skills.

**Conclusions:**

Effective clinical instructors have a specific characteristics identified by student. The most important characteristics of effective clinical instructors were related to communication and teaching skills, internal motivation, and professional appearance. More research is necessary to determine the relationship between the characteristics, instructor competency, and student learning outcomes.

**Supplementary Information:**

The online version contains supplementary material available at 10.1186/s12912-021-00556-9.

## Background

Clinical education is the heart of nursing education [1] and the costliest part of the nursing curriculum [2]. Clinical education is a part of health care education which is provided to students by experienced clinical instructors in educational and medical centres such as hospitals and outpatient clinics [3]. Evidence suggests that clinical instructors are the most important factor in achieving clinical outcomes [4, 5]. An instructor can compensate for the lack of textbooks and educational facilities, or turn the best learning environment and subject into a passive environment through their inability to make the desired emotional connection [4–6].

Effective clinical instructors demonstrate commitment, internal motivation, problem solving skills, flexibility and creativity, time management, leadership and management, accessibility, lifelong learning, morality, and up-to-date knowledge and skills [7]. Research suggests that the clinical instructors’ characteristics such as teaching ability, behaviour, and character affect students’ learning [4, 5, 8]. Today, the use of effective clinical instructors has become a major concern for the academic education systems [4, 9].

One method used by universities to define an effective clinical instructor is to ask students for their perspective [10]. Understanding the student perspective can have a positive effect on learning in clinical settings [11, 12].

However, there are few studies reported in the literature that have examined the characteristics of an effective clinical instructor from the student perspective. These studies note good communication and teaching skills are important [5, 13, 14]. In addition, excellent literacy, enthusiasm for education, clinical competence, and non-judgmental disposition have been noted as important characteristics of a good clinical instructor [7, 15]. The purpose of this study was to understand the student perspective about the characteristics defining an effective clinical instructor.

## Methods

### Study design

This was a descriptive qualitative study.

### Participants and sampling method

The study population was nursing students at Imam Reza Hospital in Kermanshah-Iran. The participants included twelve nursing students who were selected purposefully. The sample size was determined by data saturation. In-depth semi-structured interviews were conducted with the nursing students. The inclusion criteria were being an undergraduate or postgraduate nursing student, having passed at least one clinical course, and being willing to participate in the study. The participants were selected among the undergraduate and postgraduate students with different ages in order to increase the range of responses for qualitative analysis. An attempt was also made to select the male and female participants with different academic performances.

### Data collection method

Data was collected by face-to-face, semi-structured interviews at Imam Reza Hospital. All interviews were conducted by the fourth author, who has a PhD in Nursing and about 25 years of clinical training experience and is proficient in qualitative research. During the interviews, a series of questions were asked, such as “What are the characteristics of a good clinical instructor?”, “What characteristics in your view can lead to the effectiveness of a clinical instructor?” Further, to clarify the responses, phrases such as “why and how” were used (supplementary file). The interviewer tried to suspend his own idea about the study topic.

The researcher tried to engage the participants and guide the interview effectively so that they would stay focused on the research goals. The researcher also tried to avoid verbal or non-verbal signs of bias to allow participants to speak freely. The interviews were conducted individually in a relaxed environment, and each interview lasted between 20 and 40 min. Interviews were continued until data saturation. Data saturation occurs when adding the next interview will not make new and distinct changes in the categories formed by the previous interviews [16].

### *Data* analysis

Data analysis and data collection were performed simultaneously. After each interview, the recorded remarks were transcribed verbatim and fed into MAXQDA 2013 [17]. Content analysis was used to interpret the content of textual data to gain a deep understanding of the concepts. Then, the explicit and implicit patterns of the content of the interviews were explained to develop meaning units from the questions. These units can include concepts and phrases or words that are categorized differently according to the content and their theoretical significance [18]. MAXQDA 2013, was used for the initial coding process. If a concept was repeated in the same or the next interview, the code was re-used. The research team met regularly to analyse the collected data and initial topics and issues.

Moreover, the classification of primary codes and their relationship with new data and the formation of the categories and subcategories were reviewed throughout the data analysis. The interviews were analysed one after the other. Some of the strategies considered in this study to prevent bias included not using words that might cause bias, asking general questions at the beginning of the interview, asking sensitive questions at the end of the interview, using indirect questions instead of direct questions, being neutral during the interview process, asking different questions with different words, and interpreting data with a clear mind. To confirm the coding process, the codes, categories, and subcategories were submitted to three nursing students outside the study, and they approved the process.

### Trustworthiness

To verify the trustworthiness of the data, four criteria of credibility, transferability, dependability, and confirmability, proposed by Denzin and Lincoln, were used [19]. To increase the credibility of the study, continuous and in-depth interviews were conducted with the participants and questions were repeated to ensure the responses. By providing a quiet and private environment and reassuring the confidentiality of the information, the researchers tried to convey a sense of safety and comfort to the participants in order to obtain true answers. The quality of content analysis was enhanced using the review of other researchers as well as the participants. In addition, memos and external checking were used to confirm the credibility of the data.

One of the limitations of qualitative studies is the lack of transferability of their results [19]. Although the main goal of qualitative research is not to generalize the results like the quantitative studies, the phenomenon was carefully studied for the purpose of the transferability of results. The results were also given to three nursing students outside the study, whose experiences were compared with the results of the current study. These students were chosen by snowball sampling method, by whom the results of the study (codes, categories, and sub-categories) were confirmed. In this study, all stages of the research were described step by step in order to be judged correctly during external analysis. The research team met regularly to verify the data and agree on the correctness and relevance of the meaning units. Moreover, the interview transcripts and codes were provided to the research team to confirm the correctness of the encoding.

### Ethical considerations

The Ethics Committee of Kermanshah University of Medical Sciences approved the present study. The objectives of the study were explained to the participants and the confidentiality of the participants’ specifications and information was ensured. A written informed consent was obtained from all participants.

## Results

Data saturation was achieved at the conclusion of twelve interviews with nursing students between the ages of 23 and 36 years old, seven of whom were female. Six of them were undergraduate and the other six were postgraduate students (Table 1). Emerging from the data were 780 codes, organized into nine subcategories and then consolidated into two categories. The major categories included “specific characteristics” and “general characteristics” and subcategories included “internal motivation”, “professional acceptability”, “clinical competency”, “teaching skill”, “clinical experience”, “values”, “being a faculty member”, “appropriate appearance”, and “communication skills” (Table 2).

**Table 1:** Participants’ characteristics

Participants	Age range (years)	Grade
First	20–30	MSc.
Second	20–30	BSc.
Third	20–30	BSc.
Fourth	30–40	MSc.
Fifth	30–40	MSc.
Sixth	20–30	BSc.
Seventh	20–30	BSc.
Eighth	20–30	BSc.
Ninth	20–30	BSc.
Tenth	20–30	MSc.
Eleventh	20–30	MSc.
Twelfth	20–30	MSc

**Table 2:** Categories and subcategories related to the nursing student perspective of an effective clinical instructor

Categories	Subcategories
Specific characteristics	• Internal motivation • Professional acceptability • Clinical Competency • Teaching skills • Clinical experience
General characteristics	• Values • Being a faculty member • Appropriate appearance • Communication skills

### Specific characteristics

An effective clinical instructor should have characteristics that affect students and increase their learning and motivation. This category involved five subcategories, including “internal motivation”, “professional acceptability”, “clinical competency”, “teaching skills”, and “clinical experience”.

#### Internal motivation

Most students believed that, the motivated, interested, and energetic clinical instructors would motivate learners to learn more. One of the participants in this regard stated: “An instructor should motivate students and encourage them to peruse the training. Many students have become interested in continuing the training because of their good and successful instructors. When I was an undergraduate student, my teachers were very motivated and up to date. That time, I was very willing to continue my education then, which I did. (Participant No. 11)”.

Another participant in this regard said: “When an instructor teaches with energy and is interest and motivated, he transfers this energy to learners. If he always complains about the nursing profession (e.g. it is not a good job, it’s hard, and its salary is low), the students would start to ask themselves why are we going to become nurse? With these conditions, we will become someone like the instructor! (Participant No. 12)”.

“A clinical instructor should have patience. There are many students, and if the instructor answers their questions one by one, it takes a lot of time and energy”. “The interest and energy of the instructors are very important”. “Some of the instructors are still disappointed for why they have not become doctors. Clinical instructors must love their job”.

#### Professional acceptability

The professional identity of clinical instructors is an important factor involved in the effectiveness of their teaching and plays an important role in the acquisition of educational facilities by hospitals, which consequently increases students’ learning.

One of the participants in this regard stated: “We had an instructor whose public relations was excellent and had a good relationship with all the personnel, and all the personnel were also respecting him. So, everybody knew me as the student of him.... When the clinical instructor has a good position in the clinic and the personnel are respectful of him, it will increase the staff’s cooperation with me and helps me to undertake more clinical care of the patients, or if I want certain equipment, it will be easier for me to get it. (Participant No. 11)”.

Another participant stated: “The respect given to the clinical instructor in the wards is very important and can affect his performance and student’s learning. (Participant No. 1)” Another participant collaborated this by adding: “If a clinical instructor has a good position in the hospital and the personnel respect him, they will work better with him and his students and thus, the learning will be easier for his students. (Participant No. 10)”.

#### Clinical competency

Clinical competence is one of the most important features of a good clinical instructor. Sufficient clinical competence regarding the subject of teaching was the main point that most participants pointed out. Further, the presence of the instructor along with students in the clinical setting and filling the gap between practical and theoretical training were very important from the perspective of the participants. For example, one participant commented on the clinical competence of a clinical instructor in a particular field: “A clinical instructor who teaches a particular module in a particular field or specialty must be fluent in that particular area, so he can transfer the knowledge to students. An instructor who has not worked in the paediatric ward or has not established IV access for infant, how can he teach practical things to students? (Participant No. 10)”.

Another participant approved this issue and stated that: “An instructor who has enough experience in a particular ward or area will be able to help students more than the one who goes to that ward for the first time. (Participant No. 6)”.

Another participant commented on the presence of instructors along with the students in the clinical setting: “In the first years that students have no experience, the instructor should accompany the students during the entire time of the training, because there may be a problem or students have a question. (Participant No. 9)”.

“In the first years when students have no experience, the instructors should accompany the students during the entire time of the training because there may be a problem or students may have a question.”

Another participant stated: “The instructors themselves should be on the first line of education, I mean they should come with us and do the works on the ward such as dressing and they should show us how to do it in a proper way, so that we can learn the correct principles. But unfortunately, many instructors leave students in the hands of nurses and do not accompany them. (Participant No. 2)”.

As for the filling the gap between practical and theatrical training, another participant stated: “I prefer my instructor to make a link between theoretical and practical topics, we read a lot of things in the classroom, and we would like to see the application of them in the bedside. But unfortunately, this is not happening in reality, and some of the things we have read in the classroom are not implemented in the practice. If the instructor could close the gap between theoretical and practical training, one can say that, he is a good clinical instructor. (Participant No. 11)”.

#### Teaching skills

Teaching skill is one of the most important features of an effective clinical instructor. The participants believed that teaching skills and clinical skills are very important for the transmission of the right content. The up-to-date information of clinical instructors, their time management skills, their information transfer skills, and correct assessment of the students were among the issues that the participants referred to.

With regard to the up-to-date information of the clinical instructors, one of the participants stated: “The knowledge of clinical instructors should be up-to-date and they should know about the latest scientific changes in their field of study. (Participant No. 5)”.

As for the time management skills, a student stated: “Clinical instructors should manage time, determine the entry and exit time at the first day for students, and do not leave the setting within those designated times. For example, some clinical instructors tell their students to be at the setting at 8: 30 am, but you see them in the car park at that time and students are wondering around in the ward. Head nurses will not allow students to work without the presence of clinical instructor, and this will waste the students’ time. (Participant No. 10)”.

Regarding the teaching method and the use of multimedia teaching aids by the clinical instructor, a participant stated: “The teaching aids in our internship is inadequate, the instructors do not use educational aids such as films and CDs. I think the use of film as teaching aid is essential. (Participant No. 1)”.

Another participant referred to the assessment ability of clinical instructors: “I wish there was no such thing as a score, but now that we have it, it should be fair and accurate. The instructors should have an indicator, and determine the assessment criteria. For example, they should score the presence and absence, relationship with patient and personnel, uniform, and educational activities, and evaluate everybody with these criteria. However, I think this is the responsibility of faculty to give a standard form to clinical instructors. (Participant No. 4)”.

#### Clinical experience

Some of the participants believed that having sufficient working experience in the clinical settings is an important factor involved in the effectiveness of clinical instructors. They also believed that the combination of clinical experience and other characteristics could increase the effectiveness of instructors. One of the participants stated: “I think the first and most important feature that a clinical instructor should have is work experience for at least 10 years in different clinical areas. (Participant No. 5)”.

Another participant said: “If an instructor would have a combination of energy and motivation like the up-to-date knowledge of a young teacher and experience of an older one, you could say that, he is an ideal instructor. (Participant No. 1)”.

Another participant stated: “The experience of working in an area should be preferred over education without experience, but only if the instructor’s knowledge is up to date. (Participant No. 6)”.

### General characteristics

Another main category was the general characteristics of clinical instructors. The participants believed that general characteristics of a clinical instructor are important in the effectiveness of his/her teaching. This category included four subcategories, including “values”, “being a faculty member”, “appropriate appearance” and communication skills.

#### Values

Most participants believed the values of clinical instructors are effective in establishing a better communication and learning. In this regard, the most important points that the participants reported were the instructor as a role model for students, the transfer of calmness and confidence to students, respect for students’ justice, politeness, and responsibility. One of the participants stated: “The instructor should be scientifically, clinically, ethically and behaviourally, a role model for students. They way that, clinical instructors treat patients and personnel is under the microscope of students, and students will repeat them in the future. (Participant No. 6)”.

Another participant stated: “The clinical instructors should be flexible, because when clinical instructors behave authoritatively, students cannot learn much, and students do not like this kind of instructor. (Participant No. 5)”.

With regard to the values of clinical instructor, another participant stated: “They should be friendly with students and make students feel comfortable with them, however, they should not be too friendly. (Participant No. 12)”.

In the fourth interview, regarding the issue of discrimination in assessment, one participant stated:“ The instructor should evaluate the students during the course of internship, and the only criteria for evaluation should be the clinical practice of student, and not anything else. A good clinical instructor should be fair. (Participant No. 1)”.

#### Being a faculty member

Some students believed being a faculty member was effective in increasing the learning of nursing students, while others believed an effective clinical instructor should not necessarily be a faculty member. One of the participants stated: “Being a faculty member is effective in the learning of nursing students. We had an instructor who was a faculty member and he was very good. Now, our best teachers are faculty members. We trust them. (Participant No. 8)”.

Another participant stated: “Those who are faculty members are excellent, but those who are normal nurses and have a bachelor’s or master’s degree cannot teach us well. (Participant No. 7)”.

One of the participants who believed that being a faculty is ineffective stated: “Clinical instructor, both the faculty and non-faculty, is no different. Now, there are many non-faculty instructors but their work is much better than those of faculty members. (Participant No. 11)”.

Another participant confirmed this issue and reported: “The clinical skill of instructor is better than being a faculty member. (Participant No. 10)”.

#### Appropriate appearance

Clinical instructors should be well dressed, have appropriate appearance, and use an ID card because students are influenced by their behavior. One of the participants said: “If the instructors have appropriate appearance, the students will view them batter and their teaching will be more effective. (Participant No. 3)”.

Another participant stated: “The appearance of clinical instructors should be defined by the university. (Participant No. 9)”.

A participant confirmed the effect of instructor’s appearance on students and stated: “There is an old saying that says what you prefer for yourself prefer for others too. A female instructor who criticizes a student over her makeup should not wear makeup herself. When I see a clinical instructor does not have an appropriate appearance, for example, does not have ID card, or uniform, or have makeup on and at the same time she wants me to have all of them, I become doubtful and confuse. (Participant No. 11)”.

#### Communication skills

Most of the participants considered the clinical instructors’ communication skills to be an important factor in their effectiveness. Students’ adequate knowledge, creating a positive and effective communication with the student, and familiarity with the society’s culture and its official language were the factors that the participants reported as important communication skills. One of the participants stated: “Clinical instructor should have such a good relationship with the students, that students can easily ask questions and raise their worries. (Participant No. 1)”.

With regard to the teacher-student relationship, one participant said: “A good clinical instructor should communicate well with the students and gain their trust. There are some instructors that you just love them either in terms of communication, work or knowledge. You just respect them for their behavior and knowledge. (Participant No. 6)”.

Regarding the use of official language, one participant stated: “Clinical instructors should speak a language that is the official language, and in the group, everyone can understand and do not use local languages. Our official language is Farsi and clinical instructors should not use local languages, like Kurdish, when they teach”. This participant also confirmed the teacher-student communication by saying that: “Communication is very important, I think the first session of the internship should be an introductory session so that, the teachers and students can better understand each other. (Participant No. 2)”.

As for the familiarity of clinical instructor with the society’s culture one student stated: “The fact is that an instructor should know for what society he is training these nurses. In our society with such culture, structure, etc., what exactly should a nurse do? (Participant No. 4)”.

## Discussion

The purpose of this study was to understand the perspectives of nursing students about the characteristics of an effective clinical instructor. The results indicated the characteristics of clinical instructors influenced the perspectives of the students regarding the effectiveness of their teaching. Students believed internal motivation was one of the special characteristics of an effective clinical instructor. Studies in line with the present study have also reported internal motivation as an important characteristic of a clinical instructor [14, 20]. Other researchers have reported that a good clinical instructor is willing to teach, makes the clinical learning enjoyable, is able to motivate students and get them involved in learning, provides learning opportunities for students, and makes the clinical environment attractive [14, 15, 21, 22]. Unfortunately, there is a false belief that every educated person and expert is an instructor, while such a claim is not always true, and many experts are not good instructors [20]. These factors necessitate the significance of employing motivated instructors for clinical education to support students in achievement of academic objectives.

Communication skills were considered an important behavioural characteristic of an instructor. Studies have also reported communication skills as an essential feature of a clinical instructor [4, 8, 13, 15, 20, 23, 24]. Evidence shows that teachers with good communication skills make the clinical environment attractive for the students and enhance their motivation for learning [20, 22, 25]. Teachers with good communications skills can manage the possible clinical conflicts and prevent the effect of these conflicts on the students’ learning process [20]. Appropriate teacher-student communication reinforces the students’ stress coping skills and facilitates learning [22]. Proper communication is necessary for learning and teaching, and instructors can bring about positive academic and behavioural changes in students by establishing a good communication with students.

Being a faculty member was another characteristic of clinical instructors. To achieve clinical education objectives, it is necessary to employ faculty members [10]. A nursing faculty member is someone who helps students to achieve clinical competence via various strategies. In some clinical wards, the clinical nurses are sometimes used as instructor due to the shortage of competent faculty members. The faculty members have a special position in the education system and can professionally teach the students owing to having characteristics such as interpersonal skills and academic and clinical competencies [21, 26]. To achieve the clinical education objectives, every university should have adequate faculty members and set short-term and long-term goals to hire the required faculty members.

Professional acceptability was another characteristic of a clinical instructor. Various factors are associated with the acceptability of a clinical instructor, the most important of which are a good sense of humour, good professional communication with the personnel and patients, criticizability, and honesty [20, 27, 28]. Teachers with adequate clinical acceptability can finely manage the possible clinical conflicts and facilitate the students’ learning [20]. A teacher’s clinical acceptability can make the clinical learning experiences enjoyable for the students and provide them with maximum advantage of the clinical possibilities for learning.

Consistent with previous research [20, 24, 29, 30], clinical competence is another significant characteristic of an effective clinical instructor. There is no consensus over the characteristics of instructors with clinical competence; however, characteristics such as ethical codes, updated theoretical and clinical knowledge, effective communication with students, and managerial capabilities have been proposed as important factors in this regard [9]. Inadequate clinical competence of an instructor reduces the students’ trust and disrupts student-teacher communication [25]. Considering the significance of clinical education, clinical instructors should be aware of their professional responsibility, make their attempt to enhance their theoretical and clinical knowledge, and consider the principles of professional ethics. Furthermore, they can gain more knowledge by working with their more experienced colleagues.

Teaching skills was found to be another clinical characteristic of the clinical instructors. Studies have emphasized that clinical instructors should have academic competence [13–15, 24, 25]. The most important academic characteristics of clinical instructors include being available, using new teaching methods, encouraging students, providing regular feedback, avoiding negative feedback in the presence of the patients and staff, and using objective criteria for assessment [10, 25, 31, 32]. As the main pillar of education, an instructor should have sufficient learning and teaching skills to convey complex clinical concepts to students in an organized and comprehensible manner.

The results indicated clinical instructors should have adequate clinical experience. Other studies have also reported sufficient experience as an important property for a clinical instructor [23, 24]. Evidence shows that an experienced instructor has higher self-confidence and is more efficient in achieving academic objectives [33, 34]. In this regard, there is a Persian proverb which says experience is the mother of science. If clinical instructors do not have sufficient experience, their academic status will be questioned by the students and personnel, and they will not have the required efficiency [20]. Given the significance of clinical education, adequate clinical experience should be considered while choosing clinical instructors.

Ethical orientation was considered another characteristic of a clinical instructor. Similar studies have also emphasized that clinical instructors should be good role models for their students, should have characteristics such as honesty, confidentiality, criticizability, sense of humour, and sincerity, and should encourage their students [9, 10, 20, 25, 35]. Studies have shown that a clinical instructor’s morality can make the clinical experiences enjoyable for the students [22, 36]. The ethics-oriented instructors as role models can boost the professional ethical values in students.

Appearance was another characteristic of a clinical instructor. Appearance deals with an instructor’s physical features, neatness, and clothing. A qualitative study in Iran showed the nursing students believed the physical characteristics of a clinical instructor were an influential factor involved in clinical education [37]. Another study in Iran indicated the nursing students reported having a neat and tidy appearance as the most important feature of a clinical instructor [38]. The nursing instructors as role models [21] should pay enough attention to their appearance.

### Limitations

The main limitation of qualitative studies is the lack of generalizability of the results, to which our study is not an exception. Another limitation is related to the purposeful sampling method. In this method, the samples may not be representative of the population and therefore selection bias may occur. Further, the validity of the data may be affected by the purposeful sampling method. The results may be specific to this environment.

## Conclusion

The most important characteristics of an effective clinical instructor were having intrinsic motivation, teaching skills, adequate clinical competence, professional ethics, sufficient clinical experience, appropriate communication skills, professional acceptability, and appropriate appearance and being a faculty member. When selecting clinical instructors, the schools of nursing should consider these characteristics. The faculty members are required to continuously enhance their professional competence to increase the students’ clinical learning quality. Their knowledge of the indices of an effective clinical instructor from the perspective of students can help them in promoting the quality of their teaching skills. The results of this study can be employed by the novice nursing instructors to consider the behaviours that promote students’ learning behaviours. Despite sufficient studies about the characteristics of effective clinical instructors, further studies are still needed in this regard.

## Supplementary Information

**Additional file 1.**

## Data Availability

The identified datasets analyzed during the current study are available from the corresponding author on reasonable request.
