# Supplementary material for: The characteristics of an effective clinical instructor from the perspective of nursing students: a qualitative descriptive study in Iran
Source: BMC Nurs. 2021 Mar 4;20:36. doi: 10.1186/s12912-021-00556-9 (PMC7934364; doi:10.1186/s12912-021-00556-9)
Supplement: Supplementary file 1 — Additional file 1. [file 12912_2021_556_MOESM1_ESM.docx]

**Examples of main questions**

"What are the characteristics of a good clinical instructor?"

What characteristics in your view can lead to the effectiveness of a clinical instructor?"

**Examples of probing questions**

“Please tell me more about that."

“What was the situation?”

"What do you mean by that?"

"Why do you think this characteristics of a good clinical instructor is important?"

"Could you please explain more about this characteristics? "
